# Exploring the conceptualisation and study of freebirthing as a historical and social phenomenon: a meta-narrative review of diverse research traditions

**DOI:** 10.1136/medhum-2019-011786

**Published:** 2020-05-02

**Authors:** Gemma McKenzie, Glenn Robert, Elsa Montgomery

**Affiliations:** Florence Nightingale Faculty of Nursing, Midwifery and Palliative Care, King's College London, London, UK

**Keywords:** medical humanities, sociology, pregnancy, obstetrics, history

## Abstract

Freebirthing is a clandestine practice whereby women intentionally give birth without healthcare professionals (HCPs) present in countries where there are medical facilities available to assist them. Women who make this decision are frequently subjected to stigma and condemnation, yet research on the phenomenon suggests that women’s motivations are often complex. The aim of this review was to explore how freebirth has been conceptualised over time in the English-language academic and grey literature. The meta-narrative methodology employed enables a phenomenon to be understood within and between differing research traditions, as well as against its social and historical context. Our research uncovered nine research traditions (nursing, autobiographical text with birthing philosophy, midwifery, activism, medicine, sociology, law and ethics, pregnancy and birth advice, and anthropology) originating from eight countries and spanning the years 1957–2018. Most of the texts were written by women, with the majority being non-empirical. Empirical studies on freebirth were usually qualitative, although there were a small number of quantitative medical and midwifery studies; these texts often focused on women’s motivations and highlighted a range of reasons as to why a woman would decide to give birth without HCPs present. Motivations frequently related to women’s previous negative maternity experiences and the type of maternity care available, for example medicalised and hospital-based. The use of the meta-narrative methodology allowed the origins of freebirth in 1950s America to be traced to present-day empirical studies of the phenomenon. This highlighted how the subject and the publication of literature relating to freebirth are embedded within their social and historical contexts. From its very inception, freebirth aligns with the medicalisation of childbirth, the position of women in society, the provision of maternity care and the way in which women experience maternity services.

## Introduction

Freebirth is a clandestine practice whereby women intentionally give birth without healthcare professionals (HCPs) present in countries where there are medical facilities available to assist them. Freebirthing women will accept all, some or no antenatal care, and will either inform HCPs of their plans prebirth or postbirth or disguise their freebirths as ‘born before arrivals’ (BBA), that is, the baby was born too quickly to call for an ambulance.1–3 Given the secretive nature of the practice, there are no accurate statistics on the number of women who freebirth. When estimates are given, they are often combined with precipitous labours and births4 or with BBA rates.5 Nevertheless, freebirth has become common enough to feature in the media, grey literature and more recently in empirical academic research.

While freebirth may be viewed as a decision taken by a minority of women, an exploration of the phenomenon has implications for much wider aspects of the maternity system. In essence, freebirthers are declining maternity care, and this raises questions about the type and quality of services being offered to women. While on a global level the WHO highlights that maternal mortality rates have dropped in the last 30 years,6 it has also raised concern about the medicalisation of childbirth and its consequences.7 In 2017 in England for example, only 2.1% of births took place at home,8 and between 2016 and 2017, 29.4% of labours in English National Health Service hospitals were induced,9 while 27.8% of births were via caesarean section.10 Similar trends are apparent in the USA11 and Australia.12 It is questionable what role this medicalisation has played in women’s decisions to remove themselves entirely from the maternity system.

The media frequently presents freebirth as a ‘deviant’ behaviour, and online newspaper reports often attract negative public comments whereby freebirthing women are considered irresponsible, selfish, stupid and rash.13–15 Consequently, it is a decision that gives rise to stigma and condemnation. Given that birth in Western industrialised nations is viewed as a medical event requiring the expertise of professionals, it is important to understand such non-conformist behaviour. Further, in order to ensure an appropriate policy response, it is crucial to explore whether freebirth is connected to any wider societal factors and what the consequences are for women who make this birthing decision.

It is only in the last decade that freebirth has come under academic scrutiny, and there are few empirical studies of the phenomenon. Three literature reviews have been published in midwifery journals,16–18 and each focuses on understanding women’s motivations. Although there is one review of American law,19 there are no literature reviews that have been published beyond a midwifery perspective.

The overall aim of our review was to explore how freebirth has been conceptualised over time in the peer-reviewed and grey literature. Our objectives were to understand the following:

- Women’s freebirthing motivations.
- The social and historical context in which freebirth takes place.
- How social factors may have influenced the development of research and the publication of literature pertaining to freebirth.
- How different traditions in the freebirth literature have shaped academic discourse.

## Methods

### Public and patient involvement

This review is part of a larger empirical project exploring the experiences of women who freebirth their babies in the UK. All aspects of the project are supported by AIMS (Association for Improvements in the Maternity Services), a UK national charity which assists women in navigating the maternity system. Support has included introduction to freebirthing women known to the charity, and multiple phone calls, emails and face-to-face discussions about the phenomenon and AIMS’ experience of supporting women making this decision. Funding was secured for the lead author to pursue a 3-month internship with AIMS with a view to enhancing public and patient involvement, particularly with regard to knowledge exchange between academia and the third sector. The internship provided the opportunity for the lead author to understand the phenomenon of freebirth against the wider backdrop of the UK maternity services more generally. While the internship did not directly inform the review, it did provide a more holistic insight into the phenomenon, which was useful when interpreting the existing literature. In addition, the review was complemented by interaction with freebirthing women face-to-face and online, specifically with regard to their own birthing experiences as well as their suggestions for relevant freebirthing literature.

### Definitions

For the purposes of the review, freebirth occurs when a person intentionally gives birth without a registered HCP present in a country in which there is an established state maternity system. The word ‘person’ was used instead of woman due to anecdotal reports of freebirth occurring in the transgender community. The use of the word ‘intentionally’ was to exclude people who had precipitous labours and to delineate between those who accidentally give birth alone and people who actively decide to do so. Registered HCPs are those who are either licensed, certified or regulated by the state to legitimately and legally attend women in labour and birth (ie, doctors or midwives). This precludes unregistered birth workers such as doulas, lay midwives and birth educators. Finally, the requirement that freebirth takes place in a country in which there is an established state maternity system excludes eras and places where no maternity provision is/was available. This reinforces the important point that freebirth is an active decision to step out of a maternity system.

### Meta-narrative

The goal of a systematic review is to synthesise a large body of evidence using an explicit, transparent and predetermined method. Difficulties can arise however when the body of evidence is complex, and covers a range of disciplines, methodologies and research designs.20

Meta-narrative is a form of systematic review created as a way of overcoming these difficulties and was designed primarily for ‘topics that have been differently conceptualized and studied by different groups of researchers’.21 Inspired by Kuhn’s 1962 book *The Structure of Scientific Revolutions*,22 it enables evidence to be understood within the context of a particular research tradition and its scientific developments, as well as against its much larger overarching social and historical setting. As Wong writes:

Meta-narrative review looks historically at how particular research traditions have unfolded over time and shaped the kind of questions being asked and the methods used to answer them.23

Using historical examples, Kuhn argues that scientific research is undulating and takes place within research traditions based on particular ‘paradigms’, that is, a scientific community’s shared understanding and commitment to a set of rules and standards.24 These paradigms are based on what is known and understood at the time, and research within that tradition builds on this until the emergence of a new paradigm in the form of a scientific discovery and a shift in understanding.

Meta-narrative uses these ideas as a foundation to understand and explore disparate types of evidence on a topic from a range of disciplines. This allows the trajectory of the scientific evidence to be charted and the storyline of a research tradition to unfold.25

### Inclusion and exclusion criteria

All included papers had to adhere to the definition of freebirth as described earlier. Only English-language literature was sourced. There was no limit placed on the year of publication as we wanted to understand the origins of the phenomenon. Similarly, there was no restriction placed on the country in which the birth took place, as it was important to explore where freebirth was happening at different times.

With regard to the type of literature sourced, all types of literature were included except journalism. The reason for this exclusion was that it may lead to the inclusion of blogs and social media posts. Such wide inclusion criteria would have made the amount of recent literature sourced unmanageable.

### Informal search

Following the guidance of Greenhalgh *et al*, the search phase began informally and in an unstructured way.26 Beyond midwifery, it was initially unknown which disciplines had engaged with the concept of freebirth and from which countries this literature would originate.

At this early stage literature was largely sourced based on our own prior knowledge, contact with researchers who had pursued similar work and from activists involved in AIMS. The lead author also posted a request for literature suggestions on an online freebirth group, and some ideas were given by community members who pointed us towards publications such as Carter27 and Moran.28 In addition, citation checking of collected freebirth articles led to the consideration of further research and literature; this was particularly relevant to medical studies, that is, Burnett *et al* 29 and Asser and Swan.30 Notably, it was during this phase that much of the grey literature was sourced.

### Formal search

In accordance with Greenhalgh *et al*,31 a formal search was then carried out. After consideration, four databases were included: Medline, Embase, Maternity and Infant Care Database, and Social Sciences Citation Index. The review team perceived that these databases would be the most likely to capture relevant texts on freebirth. The inclusion/exclusion criteria were as described earlier.

Although freebirth is the term used to define the type of birth explored here, there are other terms used in activism and in the literature to describe the same phenomenon. It was unclear where (geographically) various terms were most frequently used, in what context and era, and in what research tradition. All of the terms in table 1 were entered independently into each database using Boolean truncation where relevant.

**Table 1:** Terms used in formal search.

Freebirth	Unassisted childbirth	Unassisted homebirth	Unhindered birth	Autonomous birth
Undisturbed birth	Abandoned birth	Unattended birth	Do-it-yourself birth	Husband assisted birth
Solo birth	Lone birth	Intuitive birth	Couples’ birth	Pure birth
Private birth	Sovereign birth	Parent assisted birth	Unassisted birth after caesarean	Planned birth before arrival

## Results

After deduplication and citation checking, there were 365 potential inclusions. The lead author read the abstracts and made exclusions based on the criteria. One hundred and nineteen texts were sourced for full-text reading. Much of the relevant literature was either unpublished academic studies, self-published or in obscure journals. Thirty-four references had to be sourced from interlibrary loans and four of those from the Library of Congress in the USA. Twelve articles proved unobtainable, but based on their abstracts it is unlikely that these were seminal pieces.

The lead author led the analysis, but contentious texts such as Gehb *et al* 32 and Ireland *et al* 33 were read, considered and discussed by all of the research team. Finally, 75 texts were considered as satisfying the inclusion criteria and were therefore included in the review.

**Figure 1:**
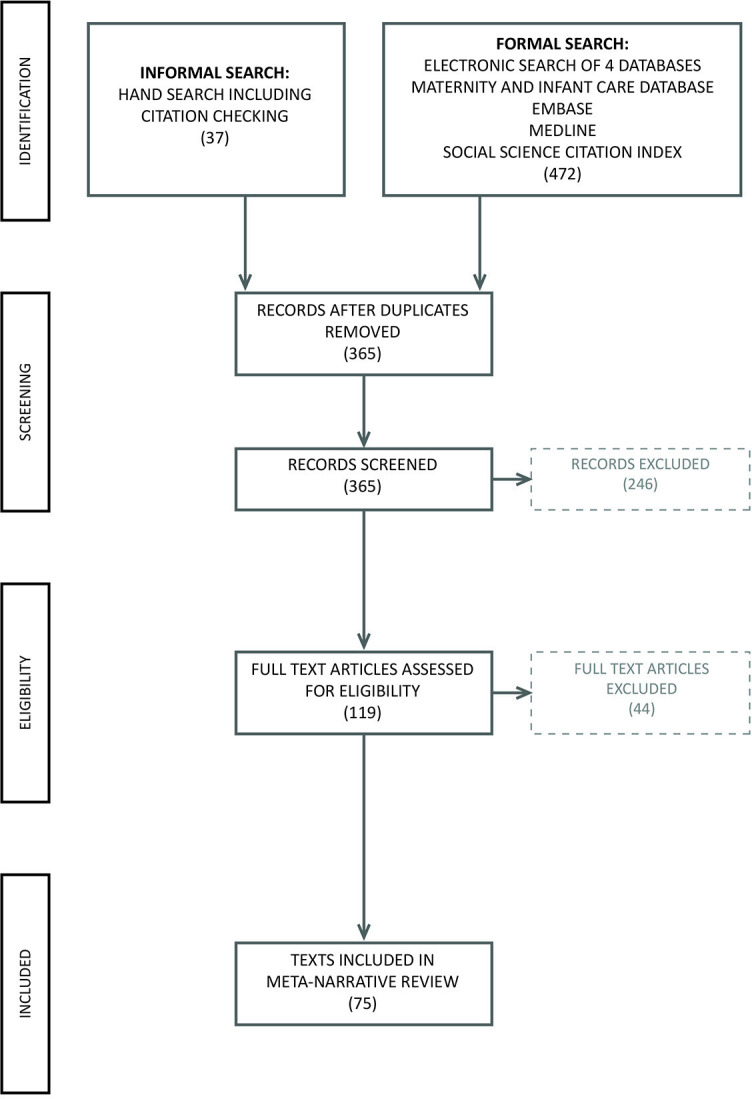
PRISMA diagram. PRISMA, Preferred Reporting Items for Systematic Reviews and Meta-Analyses.

A Preferred Reporting Items for Systematic Reviews and Meta-Analyses diagram, shown in figure 1, demonstrates the search process and its outcome.

### Overview of results

Of the 75 included texts, 27 were empirical studies and 48 were non-empirical. There was a wide range of disciplines, and the texts were categorised into nine research traditions: nursing, autobiographical text with birthing philosophy, midwifery, activism, medicine, sociology, law and ethics, pregnancy and birth advice, and anthropology. The various texts and the research traditions in which they are categorised are provided in online supplementary appendix 1.34 The dates of the included publications ranged from 1957 to 2018 and spanned eight countries and four continents.

10.1136/medhum-2019-011786.supp1 Supplementary data

Almost all of the papers were written by women, many of whom were freebirthers. The majority of literature is non-empirical; however, of the 27 empirical studies, only 5 were quantitative, 3 appearing in the medicine research tradition and 2 in midwifery. Notably the empirical study of freebirth only began in earnest post-2005.

The categorisation of literature into relevant research traditions was not complicated as most fell naturally within academic disciplines. Peer-reviewed journal publications were categorised according to their discipline of publication. For example, an article published in a midwifery journal was considered part of the midwifery tradition. Unpublished PhD texts were categorised according to the author’s university department, and MA dissertations were based on the wider discipline the author was studying, for example sociology. While autobiographical texts and activism are not scientific research traditions per se, their inclusion is important as many of the texts in these traditions have been highly influential to later academic literature and the way in which freebirth has been conceptualised.

With regard to autobiographical texts, six American women have written extensive personal narratives on freebirth which provide far more detail than any of the other texts. While the texts do not build on scientific paradigms as advocated by Kuhn, they do build on each other and share many similarities. After reading them, it was clear that they formed a particular ‘body’ of work that was very different from the other included academic texts.

Similarly, to exclude the activism literature would have led to the loss of an important conclusion that these texts informed, namely that activists were aware of and exploring the concept of freebirth, much earlier than academics. As highlighted with autobiographical texts, activism created a specific ‘body’ of work that was connected by its political motives, that is, to challenge the existing maternity system. Consequently, both autobiographical texts and activism became two separate research traditions.

### Overview of the research traditions

Table 2 provides an overview of the type of literature that appeared in each research tradition. As can be seen, nursing consists of only one study, while midwifery has provided the most literature, and this focuses largely on qualitative research exploring the lived experience. While the medical literature does include one qualitative study, its emphasis has been quantitative research on very specific populations, namely religious communities in the USA. These three research traditions are discussed in detail in the following paragraphs.

**Table 2:** Overview of the research traditions.

Research tradition	Texts (n)	Disciplines included	Types of literature	Focus
Nursing	1	Nursing	Qualitative empirical study.	One study, which focuses on the experiences of women in California in 1971 who decide to freebirth.
Midwifery	34	Midwifery	Opinion, literature reviews, qualitative empirical studies, quantitative empirical studies, narratives, academic argument, conference abstract.	Midwifery has explored freebirth in the most detail, with an emphasis on understanding the lived experiences of freebirthing women. Literature also explores the role of the maternity services and the provision of care in creating circumstances where women are more likely to freebirth their babies.
Medicine	8	Obstetrics and gynaecology, paediatrics, sexual and reproductive health, perinatal care.	Quantitative empirical studies, qualitative empirical study, opinion, commentary, editorial, conference abstract.	Quantitative studies have attempted to understand the health outcomes for women and babies after freebirths by analysing the mortality rates for women in religious communities in the USA who eschew all medical care. A qualitative study explored women’s freebirth experiences in Sweden. Opinion, commentary and the editorial linked freebirth to negative aspects of the maternity system, such as limited homebirth services.
Sociology	5	American studies, sociology.	Qualitative empirical studies.	Sociological studies were frequently framed within feminist discourse with academic debate incorporating, for example, stigma, Foucault and concepts of risk.
Anthropology	3	Anthropology, physical anthropology, women’s and cultural studies.	Qualitative empirical studies, poster presentation.	Anthropological studies were varied and included one on the prosecution of women in the USA who give birth unattended. A second study explored the freebirthing practices of Piro women in Peru, and a third challenged the biological argument that female humans are unable to give birth unassisted due to bipedalism and encephalisation.
Activism	9	N/A.	Report, editorial, narratives, opinion.	Activism literature came from one source: AIMS (Association for Improvement in Maternity Services). These texts highlighted the role of birth trauma in women’s freebirthing decisions, explored women’s narratives and highlighted the condemnation of some freebirthing women by HCPs, for example the use of social services and police involvement.
Autobiographical texts with birthing philosophy	9	N/A.	Narratives.	The detailed narratives of women’s freebirthing journeys including motivations, birth and postnatal experiences.
Pregnancy and birthing advice	3	Lay/non-biomedical advice.	Advisory texts, narrative.	Two sources discuss the benefits of freebirth, and the third describes the condemnation the author experienced while outlining his experiences as a husband of a freebirthing woman on national television.
Law and ethics	3	Law, medical ethics, ethics.	Review, student essay, academic argument.	The review explored the lawfulness of freebirth in the USA, while the academic argument explored freebirth within a wider discussion on the regulatory framework for unregulated birth workers in Australia. The student essay emphasises the need for open dialogue between pregnant women and HCPs, and the requirement that maternity services better fulfil women’s needs.

HCPs, healthcare professionals; N/A, not applicable.

Autobiographical texts with birthing philosophy provide the richest detail of women’s motivations and experiences. However, the published books on the subject are all American, thus limiting our understanding to the North American context. These texts and their authors have been highly influential in shaping the way freebirth has been conceptualised. This will be further explored below.

Activist literature presents issues from the UK that are yet to be fully explored in academia. In the two most relevant articles, Beech35 describes the case of one freebirthing family who were harassed postnatally by midwives and threatened with social services. Thomas36 also writes of her experiences of social services’ harassment, which included the involvement of the police. What these articles suggest is a lack of understanding from maternity services as to freebirth, women’s birthing rights and the limited remit of HCPs in making a woman comply with accepted maternity practice and guidelines.

Perhaps unsurprisingly, the sociological texts place women’s narratives within the context of much wider theoretical debate. Spencer-Freeze’s 2008 PhD37 study is likely the most comprehensive of all of the research on the phenomenon, while Miller38 specifically focuses on the role of stigma in freebirthing women’s experiences, and Cameron39 examines the concept of risk.

There is a paucity of research in the law and ethics research tradition and none within the discipline of bioethics, which seems unusual given the nature of the subject. Dannaway and Dietz’s40 student essay provides a useful overview of many of the ethical issues associated with freebirth. It incorporates discussion on the overmedicalisation of childbirth, the risks of birthing without an HCP, the role of informed consent and the requirement that maternity services provide care that better fulfils the needs of women.

The pregnancy and birthing advice research tradition consists of three texts, two of which are books that offer freebirth advice, one from the USA41 and one from the UK.42 The third text by Freeze43 is an account from the husband of another freebirth author (Spencer-Freeze) who describes the condemnation they both experienced when appearing on national UK television to discuss their freebirthing experiences. The paper is unusual as it is from the father’s perspective, but it also serves as a detailed insight into the stigma and condemnation parents may feel when confronted about their freebirthing decisions.

Anthropology has tackled the subject from three different angles. Falk-Smith’s44 work is particularly unique as she is exploring what has been coined the ‘obstetrical dilemma’ perceived to be caused by the evolution of bipedalism and encephalisation, and the consequential notion of obligate midwifery as argued by Trevathan.45 While her research is still ongoing and her PhD on the subject not yet complete, her current arguments draw on a body of feminist literature pertaining to both anthropology and biology which explores whether these sciences are imbued with androcentric biases.46

## Discussion

Here we discuss five texts in detail; each has been influential in the way freebirth has been understood and conceptualised over time, and highlights how freebirth and the publication of texts on the subject are closely linked to social and historical contexts. To do this we have also drawn on wider relevant literature that demonstrates the influence of the included texts, and where relevant the historical backdrop against which they were published.

In addition, we also discuss the emergence of two more recent research traditions—midwifery and medicine—as these have become increasingly influential in shaping how freebirth is conceptualised. This analysis will demonstrate how each research tradition contributes its own perspectives and methodological approaches to the storyline of the research, thus shaping our understanding of the phenomenon in a very specific way.

### Origins of the freebirth literature

One of the most important conclusions to be drawn from the first empirical and non-empirical texts is that, taken together, they largely incorporate all that scholars will subsequently ‘discover’ in empirical studies decades later. Interestingly, both sources have largely been forgotten by mainstream academic studies.

#### The first non-empirical source: Patricia Cloyd Carter, *Come Gently, Sweet Lucina*, 1957 (autobiographical text with birthing philosophy)

In 1957 Patricia Cloyd Carter published the first text on freebirth. It combines her birthing philosophy with her own freebirthing experiences. Written in an era before there were any major developments in women’s reproductive rights, such as the invention of the pill and the legalisation of abortion, Carter is pioneering not only as previous childbirth books were largely written by men, but her text also contains many of the arguments and themes that appear in later literature.

As the first person to write about the subject, and as someone who is not writing within an academic discipline, many of her points do not include the terminology that present-day authors would use. For example, the term ‘freebirth’ had not yet been coined; therefore, it does not appear anywhere in the text. Instead, Carter uses the more cumbersome term ‘Euthagenesis’,47 which perhaps unsurprisingly does not gain any traction in later literature. Carter also discusses ideas that academics would decades later crystallise into recognised sociological concepts. In her book, however, these ideas are still quite nebulous and not fully articulated.

An example of such an idea is the psychological impact of birth trauma. Not yet coined as a term nor fully recognised or understood, Carter alludes to birth trauma throughout the book and describes her own traumatic hospital births.48 She implicitly links previous birth trauma to freebirth, and even provides an example of a letter she received from a woman who was motivated to freebirth after the ‘horror of two hospital deliveries’.49 Regardless of Carter’s insight, it took over 50 years before the connection between birth trauma and freebirth was recognised in empirical studies.50

Medicalisation is another concept that Carter explores. She writes from a period in which childbirth was becoming heavily pathologised and obstetrics had begun to wield greater control over women’s birthing experiences. With a similar historical trajectory to that of the UK, childbirth in 1950s America had only recently become hospital-based. During Carter’s lifetime, hospital births in the USA had jumped from 36.9% in 1935, to 96% by 1960.51

This period was also one in which a debate rumbled over how much control a woman should have during childbirth. In 1914, twilight sleep had been introduced to the USA. This practice referred to the administration of the drug scopolamine to labouring women so as to induce amnesia. As it was not an anaesthetic, women experienced the pain of childbirth, but they did not remember it. Women were often tethered to beds to control their thrashing or placed in ‘bed-cribs’ to restrain them. In her article on the subject, Walzer Leavitt52 argues that women demanded scopolamine as a way of regaining control over the birthing process,53 that is, by deciding how they gave birth. The management of pain via this form of medication was still in use when Carter was writing. Indeed, it was used up until the 1960s.54

The relevance of this to Carter’s work is that it was published during the beginning of an alternative feminist approach to control during birth, namely natural childbirth. In 1933 and 1942, Grantly Dick-Read published two influential books, *Natural Childbirth* 55 and *Childbirth without Fear*,56, 57 respectively. Hanson argues that Dick-Read’s work, which challenged the highly medicalised approach to birth, was the start of the natural childbirth movement.58 While challenging some of his ideas, Carter draws heavily on Dick-Read’s philosophy, as do later freebirth writers.59–61 Carter’s arguments addressing the medicalisation of childbirth make reference to problems associated with the lithotomy position,62 the administration of silver nitrate into newborns’ eyes,63 the use of enemas64 and rectal examinations,65 and she likens the maternity system to a ‘packaging plant’.66 Long before feminist academics such as Oakley,67 Martin68 and Davis-Floyd69 argued that obstetrics treats female bodies as machines, Carter states that women are treated as robots as if no one realises that there are people attached to ‘this reproductive apparatus’.70 Further, she recognises the imbalance in authority between the obstetrician’s knowledge of birth and that of the mother’s, of which she states facetiously ‘is only first-hand and real’.71 Decades later, feminist scholars would write about this paradox, exploring it within the realms of authoritative knowledge,72, 73 others directly linking it to freebirth.74–77 More broadly, Foucauldian scholars would recognise this as the concept of power-knowledge, and researchers of freebirth have later explored it as such.78–81

Of equal interest is Carter’s experience of stigma. She appears to have been vocal in her birthing decisions and to have become a minor celebrity as a result.82 However, this led to some condemnation, and she writes that ‘she cannot but weep over some of the cruel letters I have received’.83 She provides an example of such a letter in which she is broadly described as a shameless, ignorant fool who is a disgrace to all women.84 Of relevance here is the general stigmatised view of freebirth and society’s response to this type of birthing decision, which was only captured in the academic literature much later.85–90 Of note is that from the 21st century onwards, the literature begins to include less puerile forms of condemnation, and instead documents police and social services intervention.91–96

#### The first empirical source: Margot E Edwards, *Unattended Home Birth*, 1973 (nursing)

In 1973, Edwards published her 1971 survey of 18 primiparous freebirthing women from the Big Sur and Santa Cruz areas of California.97 She begins by positioning herself in the research and explaining her role as a birth educator who provides antenatal classes to expectant parents. Similarly to Carter, Edwards does not use the word freebirth as the term has not yet been coined. Instead she uses the phrase unattended homebirth.

Importantly, Edwards’ study is published in 1973, the same year as *Roe v Wade*, the landmark decision of the US Supreme Court which legalised abortion in America. Reported to be the most well-known US Supreme Court decision of the 20th century,98 it is the most important legal case regarding American women’s reproductive rights. In their article on the subject, Greenhouse and Siegal highlight that the feminist and women’s rights movement played an active role in the campaign, which included nationwide marches, rallies, strike action and considerable media coverage.99 The abortion debate also extended into feminist challenges regarding the rights and roles of women in society more generally.100 What makes Edwards’ paper so interesting is that it is against this backdrop that she publishes the first empirical study of freebirth.

Of further significance is that the article was published in a nursing journal, which at first glance would set it apart from all of the other included literature. However, this reveals more about the maternity system in the USA as opposed to a unique academic perspective on freebirth. Midwifery was not—and is still not—a recognised profession in the USA. Indeed, Edwards highlights that nurse-midwifery was illegal in California at the time.101 The result is that while the USA has produced much of the freebirth literature, none of the American empirical studies is based in the midwifery research tradition.

One consequence of a lack of a recognised midwifery profession is the emergence of unlicensed midwives. Edwards highlights that these are ‘mostly self-trained lay persons’102 who provide antenatal care and attend births. While this may be presumed to be a problem of the past, the role of the unregulated birth worker (UBW) and their attendance at freebirths reappear in the most recent Australian and Canadian literature.103–106 Australian authors indicate that the lack of appropriate midwifery homebirth services motivates women to seek alternative forms of support, such as doulas to be the sole ‘professional’ in attendance during birth.107 Equally, in the Canadian research, midwifery ‘was not a licensed profession in the jurisdiction’ of the study.108 What therefore becomes apparent is that in both the earliest and most recent studies, the lack of appropriate midwifery services has been recognised as a factor in women’s decisions to have UBWs at their births as opposed to regulated HCPs.

Linked to this is the rurality of some women’s homes. Edwards notes that those relying on UBWs are often geographically isolated.109 Again, this insight appears decades later with regard to the centralisation of maternity services in Canada.110 While not explicitly challenging the maternity services on offer in 1970s America, by referencing these nuances Edwards is inadvertently highlighting the complexity of the backdrop against which women make the decision to freebirth. This complexity is not explored again empirically until over 30 years after her original publication.

Although Edwards does allude to issues within the maternity system, the focus of her study is the freebirthing women themselves. Her discourse highlights how for many couples unattended homebirth is an event in ‘an anti-establishment way of life’.111 She outlines that these women are living the ‘hip life-style’112 and notes how many are unmarried or are living in a ‘free union’.113 Others are living ‘communally’,114 which all suggests that her cohort are part of an alternative lifestyle. Although this has not been explored fully in the later literature, other authors have noted some freebirthers’ non-mainstream views and behaviour, for example cosleeping,115, 116 homeschooling,117 use of complementary medicine118 and being reactive to other forms of institution.119, 120 Removing oneself from mainstream society also appears in some American freebirth literature, notably in a spiritual sense121–123 or a geographical one.124

While Edwards does mention that some women perceive hospital treatment as ‘disrespectful and dehumanizing’,125 uniquely her cohort are all first-time mothers. Consequently, the argument that is employed in later literature that a previous bad maternity experience and/or birth trauma is a factor in freebirth does not feature.

Although there is no explicit methodology section, Edwards’ cohort includes women who intended to freebirth, but for whatever reason ended up giving birth in hospital. In her study, Edwards indicated that 11 of the 18 women did not successfully freebirth,126 which with the exception of Spencer-Freeze127 is an insight unavailable in later research. Such a focus on positive outcomes could be considered a criticism of later freebirth texts. With the exception of Griesemer128 and Spencer-Freeze,129 there are no personal narratives of freebirths gone awry, and only quantitative studies in the medical literature relate to this.

### Coining the term ‘freebirth’

In a similar vein to Carter, Parvati Baker130 published the first edition of her book *Prenatal Yoga and Natural Childbirth* in 1974.131 The book consists of yoga advice, combined with the author’s personal views and experiences of childbirth. In a pattern arising in other autobiographical texts,132–134 the author experienced a hospital birth, before later having a homebirth, and finally two freebirths.

Parvati Baker claims to have been the person to have coined the term freebirth.135 When exactly she created the term is unclear. Griesemer sheds some light on this when quoting Parvati Baker’s earlier work, *The Possible Family: Little House on the Edge of the Millennium* (1995).136 Griesemer137 quotes Parvati Baker as writing:

Freebirth is giving birth in fullest freedom without paying anyone to be paranoid for you. There are no costs at any level as what is valued is core responsibility, rather than buying someone else to take on this primal opportunity to cultivate responsibility.

In Parvati Baker’s view, therefore, the term ‘free’ relates not only to a psychological mindset, but also to a financial situation. While in the USA maternity provision comes with a price tag, from a UK perspective this financial element of the term has less relevance.

Notably, Parvati Baker was a childbirth activist and conference speaker; therefore, she may have been using the term orally long before 1995. Nevertheless, this is likely to be the first published reference to freebirth and an explanation as to what it is. Although the original text is now unobtainable, the term has gained momentum enough to be used in much of the following literature over the next several decades.

In 1994 and within the same research tradition, Laura Shanley published the first edition of her book *Unassisted Childbirth*.138 The alternative term ‘unassisted childbirth’ or UC has also gained momentum, and along with freebirth it is these two terms from these two authors which feature most heavily in the later literature.

### The notoriety of *Born in Zion*

Within the same research tradition but writing from a very different perspective is Balizet,139 the founder of Zion birth ministries and a non-freebirther who attends other women’s freebirths in a religious capacity. The first two editions of her book were unobtainable (1992 and 1994), and the third edition had to be sourced from the Library of Congress in the USA. The difficulty in sourcing the book may be due to its content and influence.

Not always considered part of the freebirth literature,140 Balizet stands far beyond the freebirth mainstream due to the author’s rejection of all medical intervention relating to both birth and illness, and her Christian belief in the power of prayer to resolve medical emergencies. Notably, in none of the other literature are her views and so called ‘Zion Births’ advocated, nor the rejection of all medical care promoted. In other autobiographical texts, writers are quick to highlight that freebirth is simply one legitimate option.141

Ironically, however, Balizet is a former nurse, and while prayer is her obstetric instrument of choice she does not remove herself entirely from the mainstream biomedical model of childbirth. Throughout the book there are references to her using a suction bulb to clear the baby’s airways immediately postbirth,142 weighing the newborn,143 using mouth-to-mouth resuscitation144 and manually extracting a baby from its mother (although on God’s instruction).145 Conversely, some of her views would not enter the biomedical paradigms. Examples of how Balizet believes labour can become slowed include the presence of a nearby nudist camp,146 and ornaments of frogs and owls in a woman’s home.147

Nevertheless, Balizet does share parallels with other freebirth literature. While religion is Balizet’s main focus, religion and spirituality do appear as lesser factors in other texts.148–151 Balizet also argues that fear affects birth.152 This aligns with Dick-Read’s influential work, which appears in many of the freebirth texts. In Balizet’s unique view, however, this fear is caused by a battle between God and Satan.

Although this work stands in contrast to its contemporaries, it has had some influence and has gained notoriety, due to its links to Christian fundamentalism and to a number of deaths. In his text *When Prayer Fails: Faith Healing, Children and the Law*, Peters dedicates a full chapter to exploring the legal cases pertaining to three members of a small religious denomination in Massachusetts known as The Body (also colloquially deemed the Attleboro Cult).153

‘Profoundly influenced’ by the teachings of Carol Balizet, the community eschewed all medical care.154 As a result, in 1999 a member of this community, Rebecca Corneau, freebirthed her son Jeremiah, who died at birth. The body was then buried in a local park alongside the remains of 10-month-old Samuel Robidoux, whose parents Karen and Jacques had starved to death based on an instruction from God.155 The cases drew national media attention. Jacques Robidoux was convicted of first-degree murder, and his wife exonerated ‘with her attorney arguing that she was a psychologically battered woman who had been victimized by a “bizarre, misbegotten group”’.156

Of most relevance however is that Corneau, who at the time of the proceedings was pregnant, was taken into state custody to ensure she gave birth with medical professionals present. This spawned considerable debate both within and outside of academia, raising legal, philosophical and bioethical questions,157 with legal scholars challenging the constitutionality of incarcerating pregnant women based on the risk of them committing a future crime.158, 159 Following later similar state incarcerations (unconnected to Balizet’s teachings), this reflects a growing trend in American scholarship to explore the parameters of state power in protecting an unborn fetus.160–163

Regardless of this notoriety, Balizet’s teachings remain in circulation. Her philosophy has been linked to the more recent Quiverfull movement in the USA.164 This movement espouses female submission to men, and the rejection of contraception in favour of pronatalism and large families.165 While freebirth has been linked to feminist discourse,166 this antifeminist stance is apparent in both Balizet and in the earlier work of Moran,167 highlighting that freebirth has not always been—nor does it always continue to be—rooted in typical feminist thought.

### The midwifery research tradition

As the autobiographical freebirth literature reached its apex in the 1990s, an alternative research tradition came to the fore: midwifery. The earliest UK paper sourced was an opinion piece published in 1997,168 and the first narrative article appearing in the midwifery press was written by the husband of a freebirthing woman.169

In 2006, the first midwifery-based empirical study that includes freebirth was published. Kornelson and Grzybowski carried out an exploratory qualitative study of 44 women living in four rural communities in British Columbia, Canada. The aim of the study was to understand ‘the realities of maternity care faced by rural women’,170 particularly as the authors note that since 2000 there had been a significant decline in the number of Canadian rural communities offering maternity care. While not about freebirth per se, the cohort included three women who had freebirthed as a response to their reality of living rurally and ‘a lack of alternatives’.171

There are two points to note here. First, this empirical study is very different from the autobiographical texts. In combination with these, it begins to highlight a spectrum on which freebirthing women sit with regard to decision making. On one end are autobiographical authors such as Shanley who make a positive choice to freebirth, and on the other end are those women who, as Kornelson and Grzybowski note, due to various circumstances feel they have no other option.

The second point is that Kornelson and Grzybowski found that rural women who were expected to leave their communities to give birth often experienced psychological and financial consequences. In order to overcome this, they developed strategies—such as freebirth—as a form of resistance. In exploring this idea, they acknowledge the position of indigenous women. While it is unclear whether any of the freebirthers in the cohort were Aboriginal Canadians, this unique perspective is acknowledged in Australian freebirth literature, in particular with regard to Aboriginal women wanting to give birth ‘on country’.172 Similar points were raised in the anthropology research tradition with regard to indigenous women in Peru who freebirth for cultural reasons.173 Such arguments indicate the heterogeneity of women who freebirth and suggest that, in attempts to support these women, some maternity systems may face additional challenges that are not present within the UK.

From 2006, there was a noticeable increase in empirical studies pertaining to freebirth. It is unclear why this is so. However, around this time within academic discourse, the concept of birth trauma evolved from the idea of it being physical trauma that occurred during birth, to one which incorporated a woman’s psychological response to her birthing experience.174 Beck’s 2004 qualitative study of 40 women’s birth experiences and her argument that clinicians viewed their trauma as routine is the start of a body of work in which the psychological aspects of birth trauma have been explored empirically.175–177

As researchers began to explore the psychological impact of birth trauma, the phenomenon began to appear within the empirical freebirth literature,178 but not within the parameters of midwifery until 2012.179 Why midwifery is slow to explore the topic empirically is unclear. This may be linked to the status of the profession, particularly in the USA, and potential limitations in funding and publication platforms. However, there is some evidence—although very little—of a connection with birth trauma within earlier non-empirical midwifery literature.180, 181 As highlighted earlier, the freebirth–birth trauma link was also made outside of midwifery decades previously182 and had been argued in activist literature as early as 2001.183

While midwifery was slow to recognise the link, since 2012, midwifery studies from various countries have highlighted the connection, that is, from Australia,184, 185 Canada,186 the UK187 and the Netherlands.188 Similarly, as reflected in Kornelson and Grzybowski, midwifery has recognised issues relating to the maternity system as a motivating factor in women’s decisions to freebirth, for example, its inflexibility, homebirth rules, overmedicalisation and the risks associated with hospital births.189–195 This development may reflect a methodological shift whereby the lived experience captured via qualitative research is becoming more frequently employed within midwifery, suggesting more of this information is coming to the fore.

Two UK studies stand apart from the other empirical midwifery literature, as they shift the dialogue beyond freebirthing women’s motivations. Feeley and Thomson196 explore how women navigate the maternity system when attempting to carry out their decision to freebirth. In the same year Plested and Kirkham197 published a similar study but their emphasis was on how freebirthing women experience risk and fear within the maternity system. These papers highlight the stigma and condemnation women experience when they attempt to make birthing decisions outside of the norm. In particular, these two studies illuminate tactics employed by HCPs, who attempt to make women conform to their perception of appropriate behaviour. This can be contrasted with current maternity rhetoric relating to woman-centred care and choice in England.198 Most worryingly, tactics included referrals to social services in four out of ten participants in Feeley and Thomson, and three out of ten in Plested and Kirkham. All referrals were later dropped, with Plested and Kirkham describing the referrals as being ‘deemed inappropriate’.199 Unsurprisingly, the misuse of social services referrals has been noted much earlier in activist literature.200, 201

What can be gained from these studies is the tension between a pregnant women’s reproductive, legal and human rights, and the power of the state to ‘protect’ the unborn. These are the same issues seen in the legal and bioethical arguments triggered by the consequences of Balizet’s philosophy, and in the wider relevant American scholarship. Unlike in the USA, women in the UK are not being incarcerated, but there is a similar sentiment of control and retaliation, which raises ethical questions that remain as yet unexplored within the midwifery research tradition.

### The medicine research tradition

The medical literature on freebirth can be divided into both empirical202–206 and non-empirical sources.207–209

In direct contrast to all other non-medical freebirth studies is Burnett *et al*,210 published in 1980, which serves as a fascinating insight into a biomedical analysis of freebirth at the time. This quantitative study explored the neonatal mortality rates of home deliveries in North Carolina between 1974 and 1976. Homebirths were grouped into various categories, for example, planned with a lay midwife or unplanned and precipitate. Of relevance to the present review was that the authors concluded that planned homebirths without an attendant resulted in a death rate of 30/1000 live births. In real terms this was 3 deaths out of the 100 freebirths, based on 244 544 overall births both within and outside of hospital during that time period. Notably, very little is explained with regard to the statistical power of the findings, with no discussion of confidence intervals or p values.

While these appear to be high rates, the article reveals much about the maternity system at the time. Written in an unempathetic way, the tragic deaths of some babies in non-freebirth circumstances highlight a society that displays an uncomfortable level of contempt for pregnant women and vulnerable mothers. Two babies died because ‘one mother…went to hospital but was turned away for lack of funds. The other…reportedly had been told not to go to the hospital without payment in hand’.211 Three deaths involved ‘unwed teenaged mothers charged with homicide’.212 The authors discuss two further homicides, ‘[o]ne infant was drowned in a canal and the other was grossly neglected’.213 In summarising these tragedies, the authors conclude that these ‘deliveries were judged to be either precipitate or intended without preparation for a healthy infant’,214 thus placing all blame on the mother’s decision making and none on wider society. These cases expose a sad underbelly of women either unable or unwilling to access US maternity provision.

Such tragedies raise questions about why the women who did freebirth did so. While Burnett *et al* highlight that they had a low-risk demographic profile,215 there is no qualitative exploration as to why they made this decision, nor consideration of, for example, their insurance status or socioeconomic background. This study therefore highlights the limits of a biomedical, quantitative approach to exploring freebirth, and demonstrates the importance of later qualitative studies to understanding this phenomenon.

Two further medical studies reignite the issues associated with religious denominations who eschew all medical care. Kaunitz *et al* 216 investigated the perinatal and maternal mortality rates of the Faith Assembly in north-eastern Indiana, USA from 1975 to 1982. They discovered that the group had a perinatal mortality rate three times higher and a maternal mortality rate 100 times higher than the state-wide rates. Similarly, Asser and Swan217 explored child fatalities from religiously motivated medical neglect between 1975 and 1995. Within their inclusion criteria were perinatal fatalities based on unattended homebirths. These numbered 59 out of 172 child deaths and were linked to a range of religious groups operating within a number of states. Commentary also included six maternal deaths.

While other freebirth writers have dismissed the relevance of these studies,218 they are important for the purposes of this review. It is unclear what the social circumstances of the women involved were, and the levels of autonomy and agency they were able to employ. Nevertheless, it would be unfair to automatically presume that these women were incapable of active decision making, or to suggest that their freebirth was not based on an honest belief that aligned with their religious and worldview. However different these motivations may be from the ‘mainstream’ freebirthers, and however unpalatable the mortality statistics, without evidence to suggest otherwise, these women appear to be making an active decision to step out of the existing maternity system and to freebirth. Consequently, these studies are important for a wider understanding of the phenomenon. What they also suggest is that the USA has an additional and more complex element to their freebirthing communities, which does not exist in literature from other countries and does not appear to be an issue within the UK.

### Strengths and limitations of the review

The main limitation of the review is that only English-language papers were sought. This prohibits a full analysis of the literature, particularly that pertaining to other Western industrialised nations such as Germany and France. In addition, the inclusion of a Peruvian study suggests that there may be relevant literature published in Spanish. Of particular relevance is the consideration of indigenous women’s cultural needs within South and Central America. A more thorough understanding of this could have better contextualised the literature in relation to indigenous women in Canada and Australia.

### Areas of future research

As noted at the beginning of this paper, current freebirth research has focused very broadly on women. Relevant studies published in the English language have only touched on how freebirth plays a role within the lives of very specific groups, for example those from particular indigenous or religious communities. It is unknown whether there are any trends based on, for example, ethnicity or socioeconomic background. As noted earlier, anecdotal suggestions of freebirth within the transcommunity did not materialise in the literature and therefore it is unknown whether some transmen are freebirthing.

The experiences of the partners of freebirthers are largely absent from the literature, as are the experiences of HCPs who come into contact with freebirthing women. While Feeley and Thomson219 and Plested and Kirkham220 begin to explore freebirthing women’s interactions with HCPs, the lack of perspectives from midwives and doctors means it is difficult to understand why some freebirthing women receive such a negative response. For policy and education purposes, it is important to know, for example, whether HCPs do not understand the rights of women with regard to their birthing decisions, and whether they have been appropriately taught how to support women who decline routine care.

The literature focuses heavily on good freebirth outcomes. With the exception of some insightful information provided by Spencer-Freeze,221 none of the qualitative research fully explores any negative consequences of freebirth. It is unknown whether this is because freebirthing women who experience difficulties seek help from HCPs, or whether women who do have negative experiences do not wish to speak out about their freebirths. From a quantitative perspective, the medical profession has made some attempt to understand outcomes. However, given the small number of freebirths and its clandestine nature, it would prove difficult to carry out a contemporary study as any results would likely lack statistical power.

## Conclusion

The meta-narrative methodology used in this review provides a way in which freebirth can be understood as a social phenomenon. Tracing freebirth’s initial origins in 1950s America through to present-day empirical midwifery studies highlights how the subject and the publication of literature relating to it is embedded within social and historical contexts. From its very inception, freebirth aligns with the medicalisation of childbirth, the position of women in society, the provision of maternity care and the way in which women experience maternity services.

The available literature highlights how freebirthing women are not a homogeneous group. However, what connects freebirthers is that when maternity services provide care that they find unpalatable or does not align with their worldview, they will find alternative ways to give birth. This form of resistance not only creates dilemmas for HCPs but also space for debate in a wide range of perspectives, ranging from law and sociology to anthropology and activism. While the results of this review highlight that writers and scholars are active in these areas, given freebirth’s relatively new appearance in the empirical literature, it is clear that researchers have only just begun to fully understand this phenomenon.

## Notes

1. Rixa Spencer-Freeze (2008), “Born Free: Unassisted Childbirth in North America” (PhD diss., University of Iowa).

2. Melanie Kathleen Jackson (2014), “Birthing Outside the System: Wanting the Best and Safest” (Phd diss., University of Western Sydney).

3. Claire Feeley and Gill Thomson (2016), “Tensions and Conflicts in ‘Choice’: Womens’ Experiences of Freebirthing in the UK,” *Midwifery*, 41: 16–21.

4. American College of Obstetricians and Gynecologists. (2016), “Committee Opinion No. 669: Planned Home Birth,” *Obstetrics & Gynecology* 128, no. 2: e26–31.

5. Charlene Eliza Thornton and Hannah Grace Dahlen (2018), “Born before Arrival in NSW, Australia (2000–2011): A Linked Population Data Study of Incidence, Location, Associated Factors and Maternal and Neonatal Outcomes,” *BMJ Open* 8, no. 3: e019328.

6. World Health Organization (2015), *Trends in Maternal Mortality: 1990 to 2015: Estimates by WHO, UNICEF, UNFPA, World Bank Group and the United Nations Population Division* (Geneva: World Health Organization), https://apps.who.int/iris/bitstream/handle/10665/194254/9789241565141_eng.pdf?sequence=1.

7. World Health Organization (2018), *WHO Recommendations: Intrapartum Care for a Positive Childbirth Experience* (Geneva: World Health Organization), https://apps.who.int/iris/bitstream/handle/10665/260178/9789241550215-eng.pdf?sequence=1.

8. Office for National Statistics (2019), “Birth Characteristics in England and Wales: 2017,” accessed January 10, 2019, https://www.ons.gov.uk/peoplepopulationandcommunity/birthsdeathsandmarriages/livebirths/bulletins/birthcharacteristicsinenglandandwales/2017.

9. NHS Digital (2017), “NHS Maternity Statistics 2016–17,” accessed November 9, 2017, https://files.digital.nhs.uk/pdf/l/1/hosp-epis-stat-mat-repo-2016-17.pdf, 9.

10. NHS Digital (2017), “NHS Maternity Statistics 2016–17,” 9.

11. Joyce A. Martin et al. (2018), “Births: Final Data for 2017,” *National Vital Statistics Reports* 67, no. 8: 6.

12. Australian Institute of Health and Welfare (2018), “National Core Maternity Indicators,” accessed October 25, 2018, https://www.aihw.gov.au/reports/per/095/ncmi-data-visualisations/contents/labour-birth/b5.

13. Ruth Styles (2013), “Freebirthing: Dangerous or Natural? Meet the Pregnant Mothers Giving Hospitals – and Midwives – A Miss,” *Mail Online*, accessed September 18, 2013, https://www.dailymail.co.uk/femail/article-2423814/Freebirthing-Dangerous-natural-Meet-pregnant-mothers-giving-hospitals--midwives--miss.html.

14. Joanna Moorhead (2013), “Freebirthing: Is Giving Birth without Medical Support Safe?” *Guardian Online*, accessed September 14, 2013, https://www.theguardian.com/lifeandstyle/2013/sep/14/freebirthing-birth-without-medical-support-safe.

15. Lacey Hayes, “Experience: I Had a Freebirth,” *Guardian Online*, accessed April 28, 2017, https://www.theguardian.com/lifeandstyle/2017/apr/28/experience-i-had-a-free-birth.

16. Claire Feeley et al. (2015), “A Meta-Thematic Synthesis, Part 1,” *Evidence Based Midwifery* 13, no. 1: 4–9.

17. Lianne Holten and Esteriek de Miranda (2016), “Women’s Motivations for Having Unassisted Childbirth or High-Risk Homebirth: An Exploration of the Literature on ‘Birthing Outside the System,’” *Midwifery* 38: 55–62.

18. Nadine Edwards and Mavis Kirkham (2013), “Birthing without a Midwife: A Literature Review,” *MIDIRS Midwifery Digest* 23, no. 1: 7–16.

19. Anna Hickman (2010), “Born (Not So) Free: Legal Limits on the Practice of Unassisted Childbirth or Freebirthing in the United States,” *Minnesota Law Review* 94: 1651–81.

20. Trisha Greenhalgh et al. (2005), “Storylines of Research in Diffusion of Innovation: A Meta-Narrative Approach to Systematic Review,” *Social Science and Medicine* 61, no. 2: 417–30, 418.

21. Geoff Wong et al. (2013), “RAMESES Publication Standards: Meta-Narrative Reviews,” *BMC Medicine* 11, no. 1: 20.

22. Thomas S. Kuhn (1962), *The Structure of Scientific Revolutions* (Chicago: University of Chicago Press).

23. Wong et al., “RAMESES Publication Standards.”

24. Kuhn, *The Structure of Scientific Revolutions*, 10–11.

25. Greenhalgh et al., “Storylines of Research.”

26. Greenhalgh et al., “Storylines of Research,” 481.

27. Patricia Cloyd Carter (1957), *Come Gently, Sweet Lucina* (Florida: Self-published).

28. Marylin A. Moran (1981), *Birth and the Dialogue of Love* (Kansas: New Nativity Press).

29. C.A. Burnett et al. (1980), “Home Delivery and Neonatal Mortality in North Carolina,” *JAMA* 244, no. 24: 2741–5.

30. S.M. Asser and R. Swan (1998), “Child Fatalities from Religion-Motivated Medical Neglect,” *Pediatrics* 101, no. 4: 625–9.

31. Greenhalgh et al., “Storylines of Research.”

32. Iris Gehb, Christian Rittner, and Klaus Püschel (2009), “Unassisted Childbirth or Homicide – Different Appraisals of Severe Injuries in a Newborn,” *Legal Medicine* 11: S174–5.

33. Sarah Ireland et al. (2011), “Niyith Nniyith Watmam (the Quiet Story): Exploring the Experiences of Aboriginal Women Who Give Birth in Their Remote Community,” *Midwifery* 27, no. 5: 634–41.

34. Only the most recent editions were accessed and analysed for the review.

35. Beverley Beech (2013),” Safeguarding Compliance,” *AIMS Journal* 25, no. 1.

36. Melissa Thomas (2013), “Freebirth and Social Services,” *AIMS Journal* 25, no. 4.

37. Spencer-Freeze, “Born Free”.

38. Amy Chasteen Miller (2012), “On the Margins of the Periphery: Unassisted Childbirth and the Management of Layered Stigma,” *Sociological Spectrum* 32, no. 5: 406–23.

39. Heather Jean Cameron (2012), “Expert on Her Own Body: Contested Framings of Risk and Expertise in Discourses on Unassisted Childbirth” (MA diss., Lakehead University).

40. Jasan Dannaway and Hans Peter Dietz (2014), “Unassisted Childbirth: Why Mothers Are Leaving the System,” *Journal of Medical Ethics* 40, no. 12: 817–20.

41. Moran, *Birth and the Dialogue of Love*.

42. Veronika Sophia Robinson (2008), *The Birthkeepers* (Cumbria: Starflower Press).

43. Eric Freeze (2012), “Freebirth,” *The Journal of Perinatal Education* 21, no. 4: 202–5.

44. Nicole Falk-Smith (2013), “Freebirth: An Obstetrical Dilemma or a Solo Act.” Poster presentation at the 82nd Annual Meeting of the American Association of Physical Anthropologists, Knoxville, Tennessee, April 9–13, 2013.

45. Wenda R. Trevathan (1987), *Human Birth: An Evolutionary Perspective – Foundations of Human Behaviour* (New York: Aldine de Gruyter).

46. Walrath Dana (2007), “Gender, Genes and the Evolution of Human Birth,” in *Feminist Anthropology: Past, Present, and Future*, ed. Pamela L. Geller and Miranda K. Stockett (Pennsylvania: University of Pennsylvania Press), 55–70.

47. Carter, *Come Gently*, 32.

48. Carter, 263.

49. Carter, 39.

50. Spencer-Freeze, “Born Free.”

51. Neal Devitt (1977), “The Transition from Home to Hospital Birth in the United States, 1930–1960,” *Birth* 4, no. 2: 47–58, 47.

52. Judith Walzer Leavitt (1980), “Birthing and Anesthesia: The Debate over Twilight Sleep,” *Women: Sex and Sexuality, Part 2* 6, no. 1: 157–164.

53. Walzer Leavitt, “Birthing and Anesthesia,” 160–1.

54. Walzer Leavitt, “Birthing and Anesthesia,” 138.

55. Grantly Dick-Read (1933), *Natural Childbirth* (London: William Heinemann).

56. Grantly Dick-Read (1942), *Childbirth without Fear* (London: William Heinemann).

57. The first edition was entitled *Revelation of Childbirth*.

58. Clare Hanson (2004), *A Cultural History of Pregnancy* (New York: Palgrave Macmillan), 138.

59. Zuki Abbot (2006), *This Sacred Life* (Milton Keynes: Self-published).

60. Robinson, *The Birthkeepers*.

61. Laura Shanley (2016), *Unassisted Childbirth* (USA: CreateSpace Independent Publishing Platform).

62. Carter, *Come Gently*, 42.

63. Carter, 76.

64. Carter, 262.

65. Carter, 266.

66. Carter, 243.

67. Ann Oakley (1980), *Women Confined: Towards a Sociology of Childbirth* (Oxford: Martin Robertson).

68. Emily Martin (1987), *The Woman in the Body* (Boston: Beacon Press).

69. Robbie Davis-Floyd (1992), “The Technocratic Model of Birth,” in *Feminist Theory in the Study of Folklore,* ed. Susan Tower Hollis, Linda Pershing and M. Jane Young (Illinois: Illinois Press), 297–326.

70. Carter, *Come Gently*, 269.

71. Carter, 278.

72. Carol F. Sargent and Robbie Davis-Floyd (1997), *Childbirth and Authoritative Knowledge: Cross-Cultural Perspectives* (California: University of California Press).

73. Sheila Kitzinger (2011), *Rediscovering Birth* (London: Pinter and Martin).

74. Spencer-Freeze, “Born Free.”

75. Lauren Ashley Brown (2009), “*Birth Visionaries: An Examination of Unassisted Childbirth*” (MA diss., Boston College).

76. Cameron, “Expert on Her Own Body.”

77. Jackson, “Birthing Outside the System.”

78. Spencer-Freeze, “Born Free.”

79. Brown, “Birth Visionaries.”

80. Cameron, “Expert on Her Own Body.”

81. Jackson, “Birthing Outside the System.”

82. Randi Hutter Epstein (2010), *Get Me Out: A History of Childbirth from the Garden of Eden to the Sperm Bank* (New York: Norton).

83. Carter, *Come Gently*, 93.

84. Carter, 320.

85. Spencer-Freeze, “Born Free.”

86. Freeze, “Freebirth.”

87. Miller, “On the Margins of the Periphery.”

88. Jackson, “Birthing Outside the System.”

89. Mariamni Plested and Mavis Kirkham (2016), “Risk and Fear in the Lived Experience of Birth Without a Midwife,” *Midwifery*, 38: 29–34.

90. Colm OBoyle (2016), “Deliberately Unassisted Birth in Ireland: Understanding Choice in Irish Maternity Services,” *British Journal of Midwifery* 24, no. 3: 181–7.

91. Spencer-Freeze, “Born Free.”

92. Jean Robinson (2001), “Lone Birth – Ethical Dilemmas,” *AIMS Journal* 13, no. 3.

93. Beech, “Safeguarding Compliance.”

94. Thomas, “Freebirth and Social Services.”

95. Feeley and Thomson, “Tensions and Conflicts in ‘Choice.’”

96. Plested and Kirkham, “Risk and Fear.”

97. M. E. Edwards (1973), “Unattended Home Birth,” *The American Journal of Nursing* 73, no. 8: 1332–5.

98. Linda Greenhouse and Reva B. Siegal (2011), “Before (and After) Roe v. Wade: New Questions About Backlash,” *The Yale Law Journal* 120, no. 8: 2028–87, 2030.

99. Greenhouse and Siegal, “Before (and After) Roe v. Wade,” 2044.

100. Greenhouse and Siegal, 2044–5.

101. Edwards, “Unattended Home Birth,” 1333.

102. Edwards, 1333.

103. Kate LeBlanc and Jude Kornelson (2015), “Giving Birth Outside the Health Care System in New Brunswick: A Qualitative Investigation,” *Canadian Journal of Midwifery Research and Practice* 14, no. 3: 8–15.

104. Elizabeth Rigg et al. (2015), “Not Addressing the Root Cause: An Analysis of Submissions Made to the South Australian Government on a Proposal to Protect Midwifery Practice,” *Women and Birth* 28, no. 2: 121–8.

105. Elizabeth Christine Rigg et al. (2017), “Why Do Women Choose an Unregulated Birth Worker to Birth at Home in Australia: A Qualitative Study,” *BMC Pregnancy and Childbirth* 17, no. 19.

106. Rebekah McWhirter (2018), “Regulation of Unregistered Birth Workers in Australia: Homebirth and Public Safety,” *Women and Birth* 31, no. 2: 134–42.

107. Hannah Dahlen, Melanie Jackson, and J. Stevens (2011) “Homebirth, freebirth and doulas: Casualty and consequences of a broken maternity system.” *Women and Birth* 24 (2011): 47-50.

222. Brown, “Birth Visionaries.”

108. LeBlanc and Kornelson, “Giving Birth Outside the Health Care System,” 11.

109. Edwards, “Unattended Home Birth,” 1333.

110. Jude Kornelsen and Stefan Grzybowski (2006), “The Reality of Resistance: The Experiences of Rural Parturient Women,” *Journal of Midwifery and Women’s Health* 51, no. 4: 260–5.

111. Edwards, “Unattended Home Birth,” 1333.

112. Edwards, 1335.

113. Edwards, 1334.

114. Edwards, 1333.

115. Hygeia Halfmoon (1998), *Primal Mothering in a Modern World* (California: Maul Bros. Publishing).

116. Shanley, “Unassisted Childbirth.”

117. Halfmoon, “Primal Mothering.”

118. Spencer-Freeze, “Born Free.”

119. Spencer-Freeze, “Born Free.”

120. Jackson, “Birthing Outside the System.”

121. Andrew M. Kaunitz et al. (1984), “Perinatal and Maternal Mortality in a Religious Group Avoiding Obstetric Care,” *American Journal of Obstetrics and Gynecology* 150, no. 7: 826–31.

122. Carol Balizet (1996), *Born in Zion* (Texas: Perazim House Publishers).

123. Asser and Swan, “Child Fatalities.”

124. Halfmoon, “Primal Mothering.”

125. Edwards, “Unattended Home Birth,” 1332.

126. Edwards, 1335.

127. Spencer-Freeze, “Born Free,” 219.

128. M Griesemer, Lyn (1998), *Unassisted Childbirth: An Act of Love* (Charleston: Terra Publishers).

129. Spencer-Freeze, “Born Free,” 252.

130. Jeanine Parvati Baker (2001), *Prenatal Yoga and Natural Childbirth* (Utah: Freestone Publishing).

131. The first (1974) and second editions (1986) could not be sourced, although the third edition (2001) was obtained.

132. Carter, *Come Gently*.

133. Griesemer, *Unassisted Childbirth*.

134. Halfmoon, “Primal Mothering.”

135. Parvati Baker, *Prenatal Yoga*, 98.

136. This book could not be sourced and it is unclear what its general content is; therefore, it was not considered for the current review.

137. Griesemer, *Unassisted Childbirth*, 56.

138. Laura Shanley (1994), *Unassisted Childbirth* (California: Praegar).

139. Balizet, *Born in Zion*.

140. Spencer-Freeze, “Born Free.”

141. Shanley, *Unassisted Childbirth*, xviii.

142. Balizet, *Born in Zion*, 65.

143. Balizet, 88.

144. Balizet, 88.

145. Balizet, 66.

146. Balizet, 35.

147. Balizet, 47.

148. Moran, *Birth and the Dialogue of Love*.

149. Abbott, *This Sacred Life*.

150. Robinson, *The Birthkeepers.*

151. Shanley, *Unassisted Childbirth*.

152. Balizet, *Born in Zion*, 13.

153. Shawn Francis Peters (2008), *When Prayer Fails: Faith Healing, Children and the Law* (Oxford: Oxford University Press).

154. Peters, *When Prayer Fails*, 155.

155. Peters, 156.

156. Peters, 159.

157. Peters, 162–3.

158. H.R Bower (2001), “How Far Can a State Go to Protect a Fetus? The Rebecca Corneau Story and the Case for Requiring Massachusetts to Follow the U.S. Constitution,” *Golden Gate University Law Review* 31, no. 1: 123–54.

159. Robin Power Morris (2002), “The Corneau Case, Furthering Trends in Fetal Rights and Religious Freedom,” *New England Journal on Criminal and Civil Confinement* 28: 89–121.

160. Rachel Roth (2000), *Making Women Pay* (New York: Cornell University Press).

161. Vicki Toscano (2005), “Misguided Retribution: Criminalization of Pregnant Women Who Take Drugs,” *Social and Legal Studies* 14, no. 3: 359–86.

162. Lynn M. Paltrow and Jeanne Flavin (2013), “Arrests of and Forced Interventions on Pregnant Women in the United States, 1973–2005: Implications for Women’s Legal Status and Public Health,” *Journal of Health Politics, Policy and Law* 38, no. 2: 299–343.

163. Howard Minkoff and Mary Faith Marshall (2016), “Fetal Risks, Relative Risks, and Relatives’ Risks,” *The American Journal of Bioethics* 16, no. 2: 3–11.

164. Kathryn Joyce (2010), *Quiverfull: Inside the Christian Patriarchy Movement* (Boston: Beacon Press), 163.

165. Emily Hunter McGowin (2018), *Quivering Families: The Quiverfull Movement and Evangelical Theology of the Family* (Minneapolis: Fortress Press), chapter 3.

166. Cameron, “Expert on Her Own Body.”

167. Moran, *Birth and the Dialogue of Love*.

168. Deborah Hughes (1997), “Private Birthing,” *Midwifery Matters* 73: 16–7.

169. B. Griesemer (1999), “Unassisted Homebirth: One Father’s Experience,” *Midwifery Today,* no. 51: 18.

170. Kornelson and Grzybowski, “The Reality of Resistance,” 260.

171. Kornelson and Grzybowski, 263.

172. Thornton and Dahlen, “Born before arrival in NSW.”

173. Luisa Elvira Belaunde (2000), “Women’s Strength: Unassisted Birth among the Piro of Amazonian Peru,” *Journal of the Anthropological Society of Oxford* 31, no. 1: 31–43.

174. Cheryl Tatano Beck (2004), “Birth Trauma: In the Eye of the Beholder,” *Nursing Research* 54, no. 1: 28–35.

175. Rakime Elmir et al. (2010), “Women’s Perceptions and Experiences of a Traumatic Birth: A Meta-Ethnography,” *Journal of Advanced Nursing* 66, no. 10: 2142–53.

176. Susan Ayers (2007), “Thoughts and Emotions during Traumatic Birth: A Qualitative Study,” *Birth* 34, no. 3: 253–63.

177. Susanne Peeler et al. (2018), “Women’s Experiences of Living with Postnatal PTSD,” *Midwifery* 56: 70–8.

178. Spencer-Freeze, “Born Free.”

179. Melanie Jackson, Hannah Dahlen, and Virginia Schmied (2012), “Birthing Outside the System: Perceptions of Risk amongst Australian Women Who Have Freebirths and High Risk Homebirths,” *Midwifery* 28, no. 5: 561–7.

180. Linda Hessel (2002), “More About Unassisted Birth,” *Midwifery Today,* no. 64: 34–5.

181. Laura Shanley (2002), “What Some Women Don’t Want,” *Midwifery Today* 63: 15–8.

182. Carter, *Come Gently*.

183. Robinson, “Lone Birth.”

184. Jackson, “Birthing Outside the System.”

185. Rigg et al., “Why Do Women Choose an Unregulated Birth Worker?”

186. LeBlanc and Kornelson, “Giving Birth Outside the Health Care System.”

187. Claire Feeley and Gill Thomson (2016), “Why Do Some Women Choose to Freebirth in the UK? An Interpretative Phenomenological Study,” *BMC Pregnancy and Childbirth* 16, no. 1: 59.

188. Martine Hollander et al. (2017), “Women’s Motivations for Choosing a High Risk Birth Setting against Medical Advice in the Netherlands: A Qualitative Analysis,” *BMC Pregnancy and Childbirth* 17, no. 1: 423.

189. H. Dahlen et al. (2011), “Home Birth and the National Australian Maternity Services Review: Too Hot to Handle?” *Women and* Birth 24, no. 4: 148–55.

190. Jackson et al., “Birthing Outside the System.”

191. Jackson, “Birthing Outside the System.”

192. O’Boyle, “Deliberately Unassisted Birth.”

193. Plested and Kirkham, “Risk and Fear.”

194. Feeley and Thomson, “Why Do Some Women Choose to Freebirth in the UK?”

195. Hollander et al., “Women’s Motivations.”

196. Feeley and Thomson, “Tensions and Conflicts in ‘Choice.’”

197. Plested and Kirkham, “Risk and Fear.”

198. National Maternity Review (2016), *Better Births: Improving Outcomes of Maternity Services in England – A Five Year Forward View for Maternity Care,* 2016, accessed July 3, 2019, https://www.england.nhs.uk/wp-content/uploads/2016/02/national-maternity-review-report.pdf.

199. Plested and Kirkham, “Risk and Fear,” 32.

200. Beech, “Safeguarding Compliance.”

201. Thomas, “Freebirth and Social Services.”

202. Burnett et al., “Home Delivery.”

203. Kaunitz et al., “Perinatal and Maternal Mortality.”

204. Asser and Swan, “Child Fatalities.”

205. Ingela Lundgren (2010), “Women’s Experiences of Giving Birth and Making Decisions Whether to Give Birth at Home When Professional Care at Home Is Not an Option in Public Health Care,” *Sexual and Reproductive Healthcare* 1, no. 2: 61–6.

206. Martine Hollander et al. (2016), “Why Women Want Other or No Delivery Care (Wonder Study),” *The Journal of Maternal-Fetal and Neonatal Medicine* 29, sup 1: XXV, European Congress of Perinatal Medicine: 15.

207. Lareen A. Newman (2008), “Why Planned Attended Homebirth Should Be More Widely Supported in Australia,” *Australian and New Zealand Journal of Obstetrics and Gynecology* 48, no. 5: 450–3.

208. Andrew Kotaska (2011), “Commentary: Routine Cesarean Section for Breech: The Unmeasured Cost,” *Birth* 38, no. 2: 162–4.

209. Beverley Chalmers (2011), “Guest Editorial: Shame on Us!” *Birth* 38, no. 4: 279–81.

210. Burnett et al., “Home Delivery.”

211. Burnett et al., 2743.

212. Burnett et al., 2743.

213. Burnett et al, 2743.

214. Burnett et al., 2743.

215. Burnett et al., 2744.

216. Kaunitz et al., “Perinatal and Maternal Mortality.”

217. Asser and Swan, “Child Fatalities.”

218. Spencer-Freeze, “Born Free.”

219. Feeley and Thomson, “Tensions and Conflicts in ‘Choice.’”

220. Plested and Kirkham, “Risk and Fear.”

221. Spencer-Freeze, “Born Free,” 252.
