# Supplementary material for: Exploring the conceptualisation and study of freebirthing as a historical and social phenomenon: a meta-narrative review of diverse research traditions
Source: Med Humanit. 2020 May 2;46(4):512–24. doi: 10.1136/medhum-2019-011786 (PMC7786152; doi:10.1136/medhum-2019-011786)
Supplement: Supplementary data [file medhum-2019-011786supp001.pdf]

Appendix 1: Included texts within each research tradition by year of publication

1. Nursing

Margot E. Edwards. "Unattended Home Birth." *The American Journal of Nursing*, 73, no 8 (1973): 1332-1335.

2. Autobiographical Text with Birthing Philosophy

Carter, Patricia Cloyd. *Come Gently, Sweet Lucina*. Florida: Self-Published, 1957.

Ursenbach Lamb, Barbara. "Unassisted Childbirth: a personal perspective." *Special Delivery* 17, no.4 (1994): 8-9

Balizet, Carol. *Born in Zion.* Texas: Perazim House Publishers, 1996

Griesemer, Lyn, M. *Unassisted Childbirth: An act of love*. Charleston: Terra Publishers, 1998.

Halfmoon, Hygeia. *Primal Mothering in a Modern World.* California: Maul Bros. Publishing, 1998.

Parvati Baker, Jeanine. *Prenatal Yoga and Natural Childbirth*. Utah: Freestone Publishing, 2001.

Rushford, Matthew. "Home Alone." *Mothering* no.156 (Sep-Oct 2009): 52-55.

Margulis, Jennifer. "Do-it-yourself birth." *Mothering* no. 162 (Sep-Oct 2010): 42-51.

Shanley, Laura. *Unassisted Childbirth*. USA: CreateSpace Independent Publishing Platform, 2016.

3. Midwifery

Hughes, Deborah. "Private Birthing." *Midwifery Matters* No.73 (June 1997): 16-17.

Griesemer, Bob. "Unassisted Homebirth: One Father's Experience." *Midwifery Today,* issue 51 (September 1999): 18.

Makala, Casey. "An Unassisted Homebirth." *Midwifery Today* Issue 52 (Dec 31, 1999): 24.

McCracken, Leilah. "Intuitive Unassisted Homebirth: This One's for the Babies." *Midwifery Today* Issue 55 (Autumn 2000): 34-35.

Hessel, Linda. "More About Unassisted Birth." *Midwifery Today* no.64 (2002): 34-35.

Shanley, Laura. "What Some Wome Don't Want." *Midwifery Today* no.63 (Autumn 2002): 15-18.

Gaskin, Ina May. "Some Thoughts on Unassisted Birth." *Midwifery Today* 66 (Summer 2003): 38-40.

Kornelson, Jude and Grzbowski, Stefan. "The Reality of Resistance: The Experiences of Rural Parturient Women." *Journal of Midwifery and Women's Health* 51, no 4 (2006): 260-26.

Abbot, Zuki. *This Sacred Life.* Milton Keynes: Self-Published, 2006.

Paul, John. "Husband-Assisted Homebirth." *Midwifery Today* no.89 (Spring 2009) 31, 67.

Dahlen, Hannah, Virginia Schmied, Sally Tracy, Melanie Jackson, Joanne Cummings, Holly Priddis. "Home birth and the National Australian Maternity Services Review: too hot to handle?" *Women and* Birth 24 (2011): 148-155.

Dahlen, Hannah, Melanie Jackson, Jeni Stevens, "Homebirth, freebirth and doulas: Casualty and consequences of a broken maternity system." *Women and Birth* 24 (2011): 47-50.

Kirkham, Mavis. "Rights, Responsibilities and Insurance: Changes and contradictions around homebirth. *Midwifery Matters* no.135 (Winter 2012): 17-18.

Jackson, Melanie, Hannah Dahlen, Virginia Schmied. "Birthing outside the system: Perceptions of risk amongst Australian women who have freebirths and high risk homebirths." *Midwifery* 28 (2012): 561-567.

Edwards, Nadine and Mavis Kirkham. "Birthing without a midwife: a literature review." *MIDIRS Midwifery Digest* 23, issue 1 (2013): 7-16.

Jackson, Melanie Kathleen. "*Birthing Outside the System: Wanting the best and safest*." PhD diss., University of Western Sydney, 2014.

Plested, Mavis. "Freebirth in Pursuit of Normal Birth: a quest for a salutogenic framework." *Essentially MIDRIS* 5 no.10 (Nov. 2014): 16-19.

LeBlanc, Kate and Kornelson, Jude. "Giving Birth Outside the Health Care System in New Brunswick: A Qualitative Investigation." *Canadian Journal of Midwifery Research and Practice* 14, no 3 (2015): 8-15.

Feely, Claire, Ethel Burns, Eike Adams and Gill Thomson. "A meta-thematic synthesis, part 1." *Evidence Based Midwifery* 13, no 1 (2015): 4-9.

Feeley, Claire and Gill Thomson. "Why Do Some Women Choose to Freebirth in the UK? An interpretive phenomenological study." *BMC Pregnancy and Childbirth* 16 (2016): 59.

Feeley, Claire and Thomson, Gill. "Tensions and Conflicts in 'Choice': Women's experiences of freebirthing in the UK." *Midwifery* 41 (2016) 16-21.

O'Boyle, Colm. "Deliberately Unassisted Birth in Ireland: Understanding choice in Irish maternity services." *British Journal of Midwifery* 24, no 3 (2016): 181-187.

Plested, Mariamni and Kirkham, Mavis. "Risk and Fear in the Lived Experience of Birth Without a Midwife." *Midwifery* 38 (2016): 29-34.

Holten, Lianne and Esteriek de Miranda. "Women's motivations for having unassisted childbirth or high-risk homebirth: An exploration of birthing outside the system." *Midwifery* 38 (2016): 55-62.

Rigg, Elizabeth Christine, Schmied, Virginia, Peters Kath and Dahlen, Hannah Grace. "Why do women choose an unregulated birth worker to birth at home in Australia: a qualitative study." *BMC Pregnancy and Childbirth* 17, no 19 (2017).

Emert, Tammy. "My Birth Story." *Midwifery Today*. Issue 121 (Spring 2017): 48-49.

Rigg, Elizabeth Christine, Schmied, Virginia, Peters Kath and Dahlen, Hannah Grace. "Why do women choose an unregulated birth worker to birth at home in Australia: a qualitative study." *BMC Pregnancy and Childbirth* 17, no 19 (2017).

Hollander, Martine, Esteriek de Miranda, Jeroen van Dillen, Irene de Graaf, Frank Vandenbussche and Lianne Holten. "Women's motivations for choosing high risk birth setting against medical advice in the Netherlands: a qualitative analysis." *BMC Pregnancy and Childbirth* 17 (2017): 423.

Brown, Debbie. "Rainbow Baby." *Midwifery Today* 122 (2017): 40-41.

Waechter, Marlene. "Concepts of Prenatal Care." *Midwifery Today* Issue 124 (Winter 2017): 22-23.

Davis, Elizabeth. "Prenatal Care: What Really Matters?" *Midwifery Today* Issue 124 (Winter 2017): 8-10.

Hollander, Martine, Lianne Holten, Annemieke Leusik, Jeroen van Dillen. "Less or more? Maternal requests that go against medical advice." *Women and Birth* 31 (2018): 505-512.

Thornton, Charlene Eliza and Dahlen, Hannah Grace. "Born before arrival in NSW, Australia (2000-2011): a linked population data study of incidence, location, associated factors and maternal and neonatal outcomes." *BMJ Open* (2018) 8:e019328.

O'Brien, Ciara, Chevelle Fairman, Lia Manuirirangi. "Students' perspective on informed consent – part 2." *Women and Birth* 31, Supplement 1 (Oct. 2018): S2.

4. Activism

Shanley, Laura. "Unassisted Childbirth." *AIMS Journal* 12 no.1 (2000).

Robinson, Jean. "Lone Birth – Ethical Dilemmas." *AIMS Journal* 13, no.3 (2001).

Beech, Beverley. "Safeguarding Compliance." *AIMS Journal* 25, no.1 (2013).

Williams, Vicki. "Editorial: Free from Choice." *AIMS Journal* 25, no.4 (2013).
Joy, Joanna. "My Birth Story." *AIMS Journal* 25, no.4 (2013).
Holdway, Sarah. "Grace." *AIMS Journal* 25, no.4 (2013).
Robertson, Hannah. "Family Birth." *AIMS Journal* 25, no.4 (2013).
Thomas, Melissa. "Freebirth and Social Services." *AIMS Journal* 25, no.4 (2013).
Williams, Vicki. "Welcome to the World." *AIMS Journal* 25, no.4 (2013).

5. Medicine

Burnett, Claude A., James Jones, Judith Rooks, Chong Hwa Chen, Carl W. Tyler and Arden Miller. "Home Delivery and Neonatal Mortality in North Carolina." *JAMA* 244, no 24 (December 1980): 2741-2745.

Kaunitz, Andrew, Spence, Craig, Danielson, TS., Rochat, Roger W., Grimes, David A. "Perinatal and maternal mortality in a religious group avoiding obstetric care," *American Journal of Obstetrics and Gynecology* 150, no 7 (1984): 826-831.

Asser, Seth M. and Rita Swan. "Child Fatalities From Religion-motivated Medical Neglect." *Pediatrics* 101, No 4 (April 1998): 625-629.

Newman, Lareen A. "Why planned attended homebirth should be more widely supported in Australia." *Australian and New Zealand Journal of Obstetrics and Gynecology* 48 (2008): 450-453.

Andrew Kotaska, "Commentary: Routine Cesarean Section for Breech: The Unmeasured Cost," *BIRTH* 38, no 2 (June 2008): 162-164.

Lundgren, Ingela. "Women's experiences of giving birth and making decisions whether to give birth at home when professional care at home is not an option in public health care." *Sexual and Reproductive Healthcare* 1 (2010): 61-66.

Chalmers, Beverley. "Guest Editorial: Shame on Us!" *BIRTH* 38, no 4 (December 2011): 279-281.

Hollander, Martine, Lianne Holten, Jeroen. van Dillen and Esteriek de Miranda. "Why women want other or no delivery care (wonder study)." *The Journal of Maternal-Fetal and Neonatal Medicine* 29, issue sup1: XXV European Congress of Perinatal Medicine (2016) 15.

6. Sociology

Spencer-Freeze, Rixa. "*Born Free: Unassisted childbirth in North America*." PhD diss., University of Iowa, 2008.

Brown, Lauren Ashley. ""*Birth Visionaries: An Examination of Unassisted Childbirth.*" MA diss., Boston College, 2009.

Miller, Amy Chasteen. ""Midwife to Myself:" Birth Narratives among Women Choosing Unassisted Homebirth." *Sociological Inquiry* 79, no.1 (February 2009): 51-74.

Miller, Amy Chasteen. "On the Margins of the Periphery: Unassisted Childbirth and the Management of Layered Stigma." *Sociological Spectrum* 32, no 5 (2012): 406-423.

Cameron, Heather Jean. "*Expert on her own Body: Contested Framings of Risk and Expertise in Discourses on Unassisted Childbirth.*" MA diss., Lakehead University, 2012.

7. Law and Ethics

Hickman, Anna. ""Born (Not So) Free:" Legal Limits on the Practice of Unassisted Childbirth or Freebirthing in the United States." *Minnesota Law Review* 94 (2010): 1651 – 1681.

Dannaway, Jason and Hans Peter Dietz. "Unassisted childbirth: why mothers are leaving the system." *J Med Ethics* 40 (2014): 817-820.

McWhirter, Rebekah. "Regulation of unregistered birth workers in Australia: Home birth and public safety." *Women and Birth* 31 (2018): 134-142.

8. Pregnancy and Birth Advice

Moran, Marylin A. *Birth and the Dialogue of Love*. Kansas: New Nativity Press, 1981.

Robinson, Veronika Sophia. *The Birthkeepers.* Cumbria: Starflower Press, 2008.

Freeze, Eric. "Freebirth." *The Journal of Perinatal Education* 21, no 4 (Fall 2012): 202-205.

9. Anthropology

Tsing, Anna Lowenhaupt. "Monster Stories: Women Charged with Perinatal Endangerment." In Ginsburg F and Anna Lowenhaupt Tsing *Uncertain Terms: Negotiating Gender in American Culture* (Boston: Beacon Press, 1990): 282-299.

Elvira Belaunde, Luisa. "Women's Strength: Unassisted Birth Among the Piro of Amazonian Peru." *Journal of the Anthropological Society of Oxford* 31, no 1 (2000): 31-43.

Falk-Smith, Nicole. "Freebirth: An Obstetrical Dilemma or a Solo Act." Poster presentation at the 82nd Annual Meeting of the American Association of Physical Anthropologists, Knoxville, Tennessee, April 9-13 2013.
